# Removal of Zearalenone and Zearalenols from Aqueous Solutions Using Insoluble Beta-Cyclodextrin Bead Polymer

**DOI:** 10.3390/toxins10060216

**Published:** 2018-05-25

**Authors:** Miklós Poór, Zelma Faisal, Afshin Zand, Tímea Bencsik, Beáta Lemli, Sándor Kunsági-Máté, Lajos Szente

**Affiliations:** 1 Department of Pharmacology, Faculty of Pharmacy, University of Pécs, Szigeti út 12, H-7624 Pécs, Hungary; faisal.zelma@gytk.pte.hu (Z.F.); af.zand@gmail.com (A.Z.); 2 János Szentágothai Research Center, University of Pécs, Ifjúság útja 20, H-7624 Pécs, Hungary; lemli.beata@gytk.pte.hu (B.L.); kunsagi-mate.sandor@gytk.pte.hu (S.K.-M.); 3 Institute of Pharmacognosy, University of Pécs, Faculty of Pharmacy, Rókus utca 2, H-7624 Pécs, Hungary; timea.bencsik@aok.pte.hu; 4 Department of Pharmaceutical Chemistry, Faculty of Pharmacy, University of Pécs, Rókus utca 2, H-7624 Pécs, Hungary; 5 CycloLab Cyclodextrin Research & Development Laboratory, Ltd., Illatos út 7, H-1097 Budapest, Hungary; szente@cyclolab.hu

**Keywords:** zearalenone, zearalenols, beta-cyclodextrin bead polymer, toxin removal, beer

## Abstract

Zearalenone (ZEN) is a *Fusarium*-derived mycotoxin, exerting xenoestrogenic effects in animals and humans. ZEN and its derivatives commonly occur in cereals and cereal-based products. During the biotransformation of ZEN, its reduced metabolites, α-zearalenol (α-ZEL) and β-zearalenol (β-ZEL), are formed; α-ZEL is even more toxic than the parent compound ZEN. Since previous studies demonstrated that ZEN and ZELs form stable complexes with β-cyclodextrins, it is reasonable to hypothesize that cyclodextrin polymers may be suitable for mycotoxin removal from aqueous solutions. In this study, the extraction of ZEN and ZELs from water, buffers, and corn beer was investigated, employing insoluble β-cyclodextrin bead polymer (BBP) as a mycotoxin-binder. Our results demonstrate that even relatively small amounts of BBP can strongly decrease the mycotoxin content of aqueous solutions (including beer). After the first application of BBP for mycotoxin binding, BBP could be completely reactivated through the elimination of ZEN from the cyclodextrin cavities by washing with a 50 *v*/*v%* ethanol-water mixture. Therefore, our study suggests that insoluble cyclodextrin polymers may be suitable tools in the future to deplete mycotoxins from contaminated drinks.

## 1. Introduction

**Figure 1:**
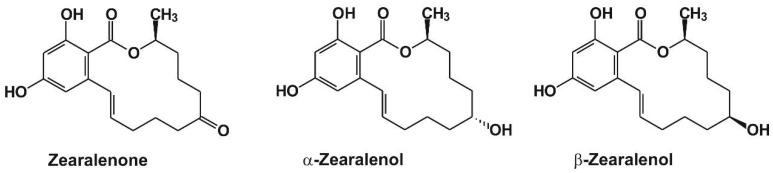
Chemical structure of zearalenone and its reduced metabolites, α- and β-zearalenol.

Zearalenone (ZEN) is a widespread mycotoxin produced by *Fusarium* fungi [1]. Despite ZEN having a non-steroidal structure (Figure 1), it is an endocrine disruptor molecule, exerting estrogenic effects in animals and humans [1,2]. After oral exposure to ZEN (through the consumption of contaminated foodstuffs), it is extensively metabolized in humans and animals, during which both phase I and phase II reactions are involved, namely the reduction of ZEN to α-zearalenol (α-ZEL) and β-zearalenol (β-ZEL) (Figure 1), and the conjugation of ZEN and ZELs with glucuronic acid [3]. Glucuronidation yields less toxic compounds [4]; however, α-ZEL is much more toxic than β-ZEL or even the parent compound ZEN, due to its higher affinity towards estrogen receptors [5,6,7]. The wide occurrence and high thermal stability of ZEN make its elimination from the food chain difficult [8]. ZEN commonly occurs as a contaminant in cereals (e.g., maize, wheat, rye, sorghum, or barley), and it also appears in spices, edible oils, milk, beer, and even in drinking water [1,9,10]. Contamination of milk and soy meal with ZELs has also been reported [11,12].

One of the most important cereal-based beverages is beer, which can be contaminated with several mycotoxins including ochratoxin A, aflatoxins, and *Fusarium*-derived mycotoxins [13]. The appearance of ZEN in beer has been described in numerous studies, showing a wide concentration range (from few nanomolar to micromolar) [14,15,16,17,18]. European and South American industrially produced beers are infrequently contaminated with mycotoxins [14]. However, home brewed South African beers commonly contain large amounts of ZEN due to the unregulated brewing procedure [15]. Furthermore, because yeasts are able to convert ZEN to ZELs [19] (e.g., during beer fermentation), lower amounts of α- or β-ZEL can also appear in some beers [20].

Cyclodextrins (CDs) are ring-shaped host molecules commonly applied in food, cosmetic, and pharmaceutical industries. The most important CDs are α-, β-, and γ-CDs, containing six, seven, and eight glucose units, respectively. The external part of CDs is hydrophilic, leading to their perfect aqueous solubility, while their internal cavity is hydrophobic, permitting CDs to accommodate lipophilic molecules or moieties [21]. As previous investigations have highlighted, β-CDs can form complexes with several mycotoxins, including aflatoxins, citrinin, ochratoxin A, and ZEN [22,23,24,25]. The stability of mycotoxin-CD complexes is highly variable. For example, whereas the binding constants (*K*) of aflatoxin B1-β-CD (*K* = 400 L/mol), citrinin-β-CD (*K* = 220 L/mol), and ochratoxin A-β-CD (*K* = 150 L/mol) complexes are relatively low, the stability of ZEN–β-CD complex (*K* = 10,000 L/mol) is more than 20 times higher [22,23,24,25]. Similarly to ZEN, its reduced metabolites, α- and β-ZEL, also form stable complexes with β-CDs [26,27]. Considering the observations that both patulin [28] and ochratoxin A [29] can be successfully extracted from aqueous solutions by β-CD polymers, it is reasonable to hypothesize that ZEN can also be removed from water or beverages through the efficient complexation of ZEN with CDs.

In this study, the removal of ZEN, α-ZEL, and β-ZEL from aqueous solutions by insoluble β-cyclodextrin bead polymer (BBP) has been tested. Because β-CD molecules were bound to insoluble beads, after their interaction with mycotoxins, CDs (together with the bound mycotoxins) are easily removable from solutions by filtration or sedimentation. First, we determined the dependence of mycotoxin removal on the time, dose, pH, and temperature, and then tested the reusability of BBP after its regeneration with 50 *v*/*v%* ethanol-water mixture. Finally, the frequent occurrence of ZEN in maize led us to investigate the suitability of BBP for the removal of ZEN from corn beer. Our results demonstrate that BBP seems a promising tool to remove even high concentrations of ZEN (and/or ZELs) from aqueous solutions, including beverages.

## 2. Results

### 2.1. Removal of ZEN, α-ZEL, and β-ZEL from Aqueous Solutions by BBP

**Figure 2:**
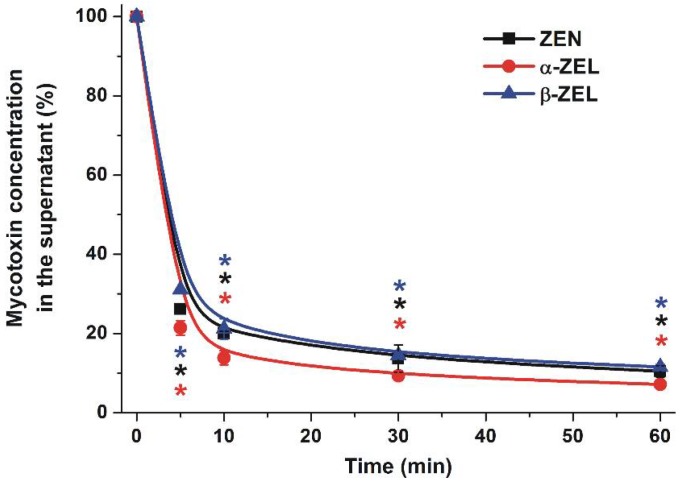
Time-dependent decreases in the concentrations (% of control) of ZEN and ZELs (10 μM each) in water in the presence of 10 mg BBP, after 0–60 min incubation periods (* *p* < 0.01).

In our first experiments, the time-dependence, dose-dependence, pH-dependence, and temperature-dependence of the removal of the mycotoxins from aqueous solutions were tested. In order to investigate the time-dependence, a relatively high amount of BBP was applied: 10 mg BBP was added to 1.5 mL of 10 μM ZEN or ZEL solutions (dissolved in distilled water), and then the samples were incubated for 5, 10, 30, and 60 min at 25 °C in a thermomixer (1000 rpm). Figure 2 demonstrates that BBP remarkably decreased the concentrations of ZEN and ZELs in aqueous solutions, leading to approximately 85–90% reduction of their mycotoxin content after 60 min incubation. Furthermore, even after a 5 min incubation, approximately 70–80% decreases in ZEN and ZEL concentrations were observed, after which the mycotoxin concentrations decreased slightly during the following 55 min. 

**Figure 3:**
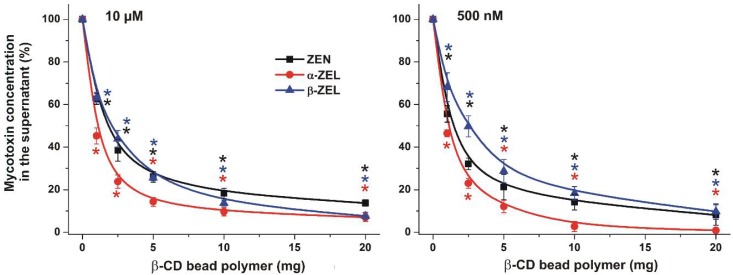
The BBP (0–20 mg) dose-dependently decreased the concentrations (% of control) of ZEN and ZELs (10 μM: **left**; 500 nM: **right**) after 40 min incubation in water (* *p* < 0.01).

Thereafter, the concentration-dependence of mycotoxin removal was examined. In these studies, the extraction of the mycotoxins was tested from solutions containing relatively high (10 μM or 3.2 ppm) and low (500 nM or 0.16 ppm) concentrations of ZEN or ZELs. The mycotoxin solutions (1.5 mL, in distilled water) were incubated for 40 min at 25 °C, in the presence of increasing amounts of BBP (0, 1, 2.5, 5, 10, and 20 mg). In a dose-dependent fashion, BBP caused significant decreases in the mycotoxin concentrations (Figure 3). In the presence of 10 μM mycotoxin concentrations, BBP was more potent in binding α-ZEL than ZEN or β-ZEL (the relative removal of the latter two mycotoxins was very similar) (Figure 3, left). In the presence of 500 nM mycotoxin concentrations, the extraction of α-ZEL was again the most effective, followed by ZEN and β-ZEL (Figure 3, right). At both initial mycotoxin levels (10 μM and 500 nM), at least 85–90% depletion of ZEN or ZEL was observed in the presence of 20 mg BBP. At 5–10 mg BBP, the removal of mycotoxins was near maximal, and higher amounts of the bead polymer only slightly reduced the mycotoxin content further.

**Figure 4:**
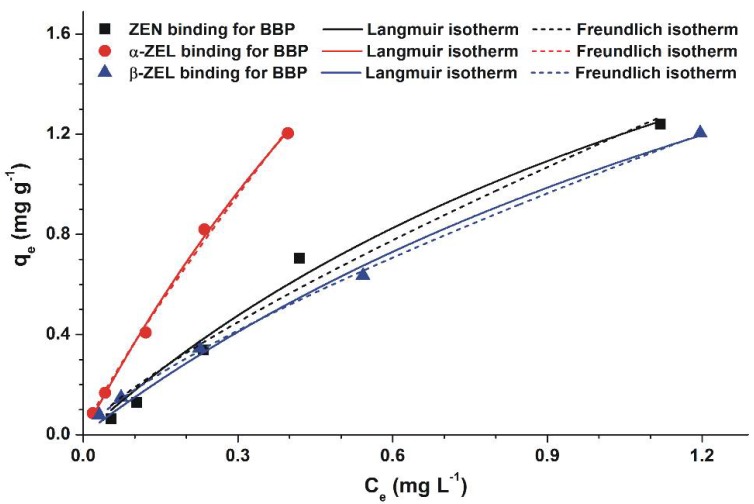
Langmuir (solid lines) and Freundlich (dashed lines) isotherms for the mycotoxin binding of BBP in water.

To investigate the mycotoxin-binding ability of BBP, increasing mycotoxin concentrations (0.5, 1.0, 2.5, 5.0, and 10.0 μM) were added to 2.5 mg BBP. After 40 min incubation at 25 °C, BBP was centrifuged and mycotoxin-content of the supernatant was determined. Figure 4 demonstrates the binding isotherms of ZEN and ZELs with BBP. Even though the Langmuir and Freundlich models are different, no preference was found between the two models under the applied conditions. Table 1 indicates isotherm parameters obtained by the graphical application of Langmuir and Freundlich models. The Langmuir equilibrium constant (*K_L_*), Freundlich constant (*K_F_*), and maximum amount of mycotoxins bound by BBP (*Q*_0_) were similar for ZEN and β-ZEL, while these parameters were much higher regarding α-ZEL. On the other hand, the heterogeneity index (*n*) was similar for each mycotoxin tested. 

**Figure 5:**
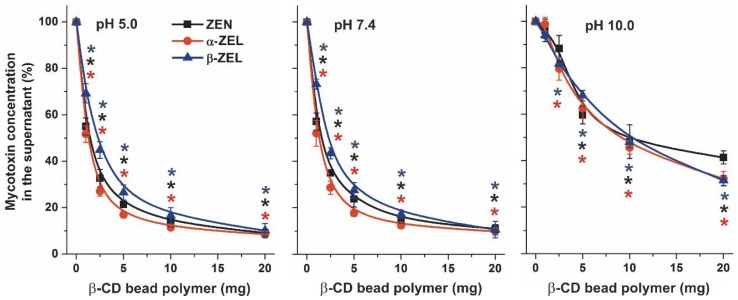
The BBP (0–20 mg) dose-dependently decreased the concentrations (% of control) of ZEN and ZELs (10 μM each) after 40 min incubation in sodium acetate (**left**; 0.05 M, pH 5.0), PBS (**middle**; pH 7.4), and sodium borate (**right**; 0.05 M, pH 10.0) buffers (* *p* < 0.01).

In order to further characterize the properties of BBP as a mycotoxin binder, the removal of ZEN and ZELs was also tested in three different buffers (pH 5.0, 7.4, and 10.0). Because ZEN and ZELs are weak acids, deprotonation (and consequently the ionization) of these molecules can influence their interaction with CDs (including BBP). Therefore, increasing quantities of BBP (0–20 mg) were added to 1.5 mL of 10 μM ZEN or ZEL solutions, dissolved in sodium acetate (pH 5.0), PBS (pH 7.4), or sodium borate (pH 10.0) buffers. As Figure 5 demonstrates, at pH 5.0 and 7.4, the extents of the removal of the mycotoxins by BBP were very similar to those seen in the previous experiment performed in water (Figure 3). However, the significantly lower toxin-binding ability of BBP was noticed for each mycotoxin at pH 10.0 than at lower pH values.

The effect of temperature on mycotoxin removal was also examined. Solutions of 10 μM ZEN (1.5 mL) with 1, 3, or 10 mg BBP added were incubated in sodium acetate buffer (pH 5.0) for 40 min at 15, 25, and 35 °C in a thermomixer (1000 rpm). Immediately after the incubations, beads were rapidly centrifuged and the supernatants were removed. After the temperature of supernatants reached room temperature, their mycotoxin contents were analyzed. Only slight, non-significant temperature-related differences were observed (data not shown).

### 2.2. Testing of the Reusability of BBP as Mycotoxin Binder, after Its Regeneration with Ethanol-Water Mixture

In the next series of experiments, the reusability of BBP was examined. After the removal of ZEN from aqueous solution, BBP was regenerated with 50 *v*/*v%* ethanol-water mixture. Then, the ZEN-extracting ability of the polymer was retested two more times (see further details in Section 4.3). Table 2 demonstrates the relative concentration (% of control) of ZEN in aqueous solution after the extraction of ZEN with BBP (row A), as well as the relative amounts of ZEN in ethanol-water mixtures after the elution of the mycotoxin from the polymer once (row B) and twice (row C). Our results highlight that after the regeneration of BBP with 2 × 1.5 mL ethanol-water mixture, BBP removed the same amounts of ZEN (92–93%) from aqueous solutions during its second and third application than at first time. Furthermore, after the two alcoholic elution steps, we completely recovered ZEN that had been extracted from the aqueous buffer by BBP.

### 2.3. Removal of ZEN from Spiked Corn Beer by BBP

**Figure 6:**
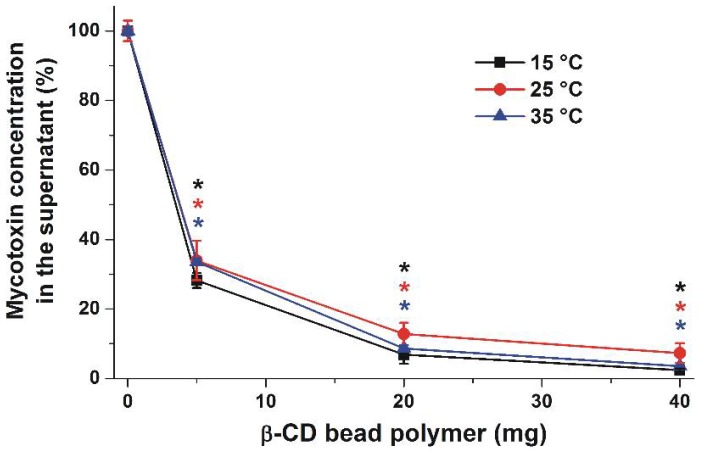
The BBP (0–40 mg) dose-dependently decreased the concentrations (% of control) of ZEN (500 nM) after 40 min incubation in corn beer at each temperature tested (* *p* < 0.01).

In order to test our hypothesis that BBP may be suitable for reducing the mycotoxin content of different drinks, the removal of ZEN from spiked corn beer samples was also investigated. It is reasonable to hypothesize that BBP may interact with other compounds in beer as well; therefore, higher amounts of the polymer (5, 20, and 40 mg) were applied in these experiments. Beer samples were spiked with 500 nM ZEN and then 1.5 mL volumes of the samples were incubated with BBP at 15, 25, and 35 °C. After centrifugation, the supernatant was removed and tempered to 25 °C, and the residual ZEN was then extracted from beer with dichloromethane. Following this, dichloromethane was evaporated, the residue was dissolved in methanol-water mixture, and ZEN was quantified by HPLC-FLD (see details in Section 4.4 and Section 4.5). ZEN in the beer samples not spiked with ZEN was not detectable. Approximately 65% of added ZEN was removed from the beer by 5 mg BBP, while more than 80 and 90% of ZEN was depleted in the presence of 20 and 40 mg BBP, respectively (Figure 6). Similarly to our previous experiment in sodium acetate buffer (pH 5.0), only slight temperature-related differences of mycotoxin-binding were observed in corn beer.

### 2.4. Effect of BBP on the Color and Polyphenol Content of Corn Beer

**Figure 7:**
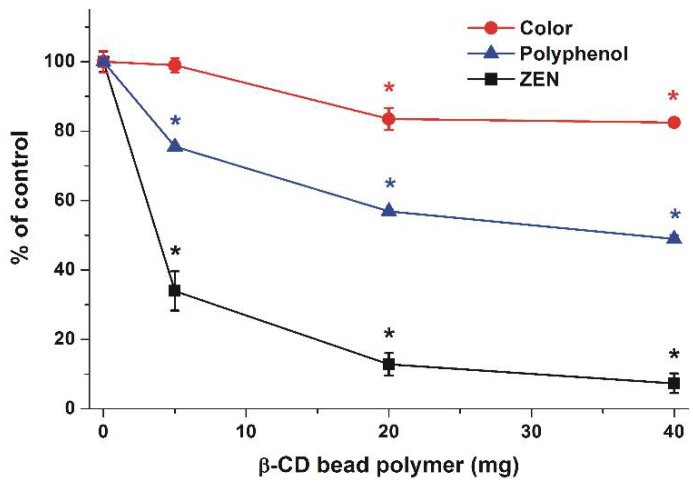
Changes of color, polyphenol content, and ZEN concentration (spiked, 500 nM) of corn beer (1.5 mL) after 40 min incubation with 5, 20, or 40 mg BBP at 25 °C (* *p* < 0.01).

Besides mycotoxins, BBP is likely able to remove other compounds from beer as well. To investigate the extent of this effect, the color and polyphenol content of corn beer (1.5 mL) were examined after its treatment with 0, 5, 20, and 40 mg BBP for 40 min at 25 °C (see details in Section 4.6). Color value (C) was 4.85 (±0.15), while polyphenol concentration (P) was 77.1 (±0.8) mg/L in controls (in the absence of BBP). As Figure 7 demonstrates, increasing amounts of BBP induced the gradual decrease of the color of beer and its polyphenol content. Even if the relative decreases in color and polyphenol content are much lower compared to the removal of ZEN, it is important to note that 20 mg of BBP caused a 16 and 43% decrease of color and polyphenol concentration, respectively. 

## 3. Discussion

As our results demonstrated, BBP rapidly interacts with ZEN and ZELs in aqueous solution (Figure 2). Thus, the mycotoxin content can be significantly decreased by incubation with BBP, even within a few minutes. Furthermore, only a few milligrams of BBP was needed to deplete the toxin in water. Interestingly, BBP caused similar relative decreases of mycotoxin concentrations in the presence of 500 nM or 10 μM of ZEN/ZELs (Figure 2), suggesting that BBP can be applied to remove these mycotoxins, even from highly contaminated samples. Previous studies indicate that usually nanomolar concentrations of ZEN are found in beverages [14,15,16,17]. However, in some extreme cases, ZEN may be present at micromolar concentrations. One of the most extreme contaminations has been reported in Zambia, where more than 14 µM ZEN was quantified in a beer sample [18]. 

The equilibrium relationship between the amounts of ZEN or ZELs bound by BBP and the quantities of free ZEN or ZELs left in the solution can be described by adsorption isotherms. There are several models to predict the equilibrium isotherm. In our study, the most commonly used Langmuir and Freundlich models were applied. These models have also been successfully applied to investigate and evaluate the sorption of ochratoxin A by the β-cyclodextrin-polyurethane polymer [29]. Similarly good fitting of Langmuir and Freundlich models occurs when the concentration of the adsorbate is small and the adsorptive capacity of the adsorbent is relatively large. Based on the Freundlich model, the heterogeneity index (*n*) is close to 1, indicating the relatively homogenous sorption of mycotoxins by BBP. Isotherm parameters clearly show the differences between the binding affinities of ZEN and ZELs towards BBP (Table 1). Both the equilibrium constant (*K_L_*) calculated by the Langmuir model and the adsorptive capacity (*K_F_*) determined using the Freundlich model support the previous observation that the interaction between α-ZEL and BBP is stronger compared to ZEN and β-ZEL. The maximum amount of mycotoxins bound by BBP was estimated based on the Langmuir model: the higher *Q*_0_ value for α-ZEL (compared to ZEN and β-ZEL) is also in accordance with the previous investigations.

The pH-dependence of mycotoxin removal is in agreement with our previous observations that CDs bind the non-ionized form of ZEN and ZELs with a much higher affinity than the ionized mycotoxins [25,27]. ZEN and ZELs are weak phenolic acids, therefore, their non-ionized forms are dominant at acidic and neutral pH, while alkaline conditions (e.g., sodium borate buffer, pH 10.0) favor the deprotonation of ZEN and ZELs, resulting in their poor interaction with β-CDs [25,27] and decreased the binding of ZEN and ZELs to BBP.

Organic solvents (including ethanol) influence the interaction of CDs with guest molecules, because they can displace the guest compound form the CD cavity. Using this principle, it was reasonable to hypothesize that after its first application for mycotoxin removal, BBP can be regenerated with ethanol or ethanol-water mixture. As it is demonstrated in Table 2, the sequestered ZEN was entirely released from BBP during the two-step washing of the polymer with 50 *v*/*v%* ethanol-water mixture, after which the regenerated BBP bound to the same extent as the unused BBP. As the regeneration of BBP is simple, CD polymers may be suitable tools in the future for the removal of mycotoxins from contaminated aqueous solutions.

After verifying the mycotoxin-binding ability of BBP in water and in aqueous buffers, the effectiveness of BBP in a complex matrix, e.g., in corn beer, was also determined. Because a nanomolar concentration of ZEN in beverages is typical, corn beer samples were spiked with 500 nM ZEN. In beer samples, 20–40 mg BBP produced a similar extent of ZEN removal (Figure 3, right) to 10–20 mg BBP in water (Figure 6), suggesting that only somewhat larger amounts of BBP are needed for mycotoxin removal from drinks than from water. Extraction of mycotoxins from beverages is complicated because different matrix components may interact with ZEN/ZELs, making their removal more difficult. Furthermore, mainly in naturally contaminated foods/drinks, mycotoxin molecules can be partly sequestered by the food matrix, resulting in these molecules possibly becoming accessible only after the digestion of the meal [30]. In addition, CDs can bind other components of drinks (e.g., polyphenols) as well, which may result in the decrease in quality of beverages. Since CDs can form (usually low-affinity) complexes with numerous compounds, the suitability of BBP to bind ZEN in beverages was questionable. However, the high stability of ZEN-β-CD and ZEL-β-CD complexes [25,27] may give some selectivity in removing these mycotoxins over other compounds naturally occurring in drinks. As it was demonstrated, BBP caused a significant decrease of polyphenol content and color of corn beer (Figure 7), suggesting the decrease of some beer constituents during mycotoxin removal. On the other hand, the relative decrease of ZEN was significantly higher compared to polyphenols. Nevertheless, we have to estimate some reduction in the quality of beer as a result of its treatment with BBP.

Despite the stability of the ochratoxin A-β-CD complex being approximately 65-fold lower than the ZEN-β-CD complex [24,25], Appell and Jackson demonstrated that the β-cyclodextrin-polyurethane polymer could significantly decrease the ochratoxin-content of aqueous solutions and red wine [29]. Patulin was also successfully extracted from apple juice by the β-cyclodextrin-polyurethane polymer for analytical purposes [27]. Besides these studies, our results further support the hypothesis that CD technology may be used for mycotoxin extraction from different drinks. Furthermore, chemical modification of the native β-CD can significantly improve its mycotoxin-binding ability: 2,6-di-*O*-methyl-β-CD binds ZEN with a six-fold higher affinity than β-CD [25], while the stability of the ochratoxin A complex with (2-hydroxy-3-*N*,*N*,*N*-trimethylamino)propyl-β-CD is approximately 200-fold higher compared to the ochratoxin A-β-CD complex [24]. Therefore, chemical modification of the native β-CD may be a promising strategy in producing more effective mycotoxin-binding polymers. Since mycotoxins are common contaminants of different drinks (including wine, beer, fruit juices, coffee, and milk), removal of these harmful compounds from beverages could be of high importance.

## 4. Materials and Methods

### 4.1. Reagents

Reagents and solvents were of analytical or spectroscopic grade. Zearalenone (ZEN), α-zearalenol (α-ZEL), and β-zearalenol (β-ZEL) were purchased from Sigma-Aldrich (Waltham, MA, USA). Insoluble β-cyclodextrin bead polymer (BBP; β-cyclodextrin-epichlorohydrin cross-linked bead polymer; CY-2011) was obtained from CycloLab Cyclodextrin Research & Development Laboratory, Ltd. (Budapest, Hungary). The polymer has a beta-cyclodextrin content of approximately 70% by weight. Stock solutions of mycotoxins (5000 μM) were prepared in ethanol (Renal, spectroscopic grade), stored at −20 °C, and protected from light.

### 4.2. Removal of ZEN and ZELs from Aqueous Solutions by BBP

In order to investigate the sorption of mycotoxins (ZEN, α-ZEL, and β-ZEL) by BBP, 1.5 mL of 500 nM (0.16 ppm) or 10 μM (3.2 ppm) mycotoxin solutions were incubated with 0, 1, 2.5, 5, 10, or 20 mg BBP in a thermomixer (1000 rpm, 25 °C). To test the time-dependence of the process, five different time periods were applied (0, 5, 10, 30, and 60 min). During the investigation of the pH-dependence of the extraction procedure, incubations were performed in sodium acetate (0.05 M, pH 5.0), phosphate buffered saline (PBS, pH 7.4), and sodium borate (0.05 M, pH 10.0) buffers. Thereafter, the samples were centrifuged (pulse centrifugation for 3 s at 4000 *g*), 500 μL supernatants were gently removed, and the mycotoxin content was analyzed by HPLC-FLD (see details in Section 4.5).

Binding of mycotoxins by BBP was also tested in the presence of a standard amount of BBP (2.5 mg) and increasing concentrations of mycotoxins (0.5, 1.0, 2.5, 5.0, and 10.0 μM), after which the results were evaluated employing the Langmuir and Freundlich isotherms. The Langmuir equation is described as [29]:

(1) $$q_{e}=\left(Q_{0}\times K_{L}\times C_{e}\right)/\left(1+K_{L}\times C_{e}\right)$$

where *q_e_* is the amount of ZEN/ZEL bound (mg) per BBP (g), while *C_e_* is the free ZEN/ZEL (mg) in the solution at equilibrium. *K_L_* is the Langmuir equilibrium constant (L/mg) and *Q*_0_ is the maximum quantity of ZEN/ZEL bound per gram of BBP. The Freundlich equation is expressed as [29]:

(2) $$q_{e}=K_{F}\times C_{e}^{\;1/n}$$

where *K_F_* is the Freundlich constant, while *n* is the heterogeneity index.

### 4.3. Regeneration of BBP and Its Reusability as Mycotoxin Binder

In order to test the reusability of BBP as a mycotoxin binder, the following experiments were performed. BBP (10 mg) was added to 1.5 mL of 10 μM ZEN solution (sodium acetate buffer, pH 5.0). After 20 min incubation at 25 °C in a thermomixer (1000 rpm), the polymer was centrifuged (pulse centrifugation for 3 s at 4000 *g*) and the supernatant was completely removed. Thereafter, the bead polymer was washed two times with 1.5 mL of 50 *v*/*v%* ethanol (20 min, 25 °C, 1000 rpm). Ethanol-water mixtures (containing the eluted mycotoxin) were removed after centrifugation (3 s at 4000 *g*). Finally, BBP was conditioned for 15 s with 1.5 mL sodium acetate buffer (pH 5.0), the polymer was centrifuged (pulse centrifugation for 3 s at 4000 *g*), and the supernatant was removed. ZEN contents of each supernatants (including the aqueous solutions and the two fractions of ethanol-water mixture) were measured by HPLC-FLD (see details in Section 4.5). 

Thereafter, the whole procedure (mycotoxin binding from aqueous solution then washing the polymer two times with 50 *v*/*v%* ethanol then once with the buffer) was repeated, after which the mycotoxin binding ability of the same polymer was tested again, applying the same conditions. 

### 4.4. Removal of ZEN from Spiked Corn Beer by BBP 

Coronita^®^ corn beer was degassed with an ultrasonic water bath and spiked with ZEN at a 500 nM final ZEN concentration. Thereafter, BBP (0, 5, 20, or 40 mg) was added to each spiked beer fraction (1.5 mL) and incubated for 40 min in a thermomixer (1000 rpm; at 15, 25 or 35 °C). At the end of the incubations, samples were centrifuged (pulse centrifugation for 3 s at 4000 *g*) and 1000 μL of the supernatants was carefully removed. The ZEN remaining in beer samples was extracted by two-step extraction with dichloromethane, during which dichloromethane (2.0 mL) was added to the supernatant (1000 μL) and the mixtures were shaken at 250 rpm for 10 min at room temperature. Then, the lower liquid phase was collected and the above described extraction was repeated with the upper (aqueous) phase. After the second extraction step, the organic phases were combined, from which the residual water was removed with anhydrous sodium sulfate. After the sedimentation of sodium sulfate, 2000 μL aliquots of the organic phase were removed and completely evaporated with a rotary evaporator (Büchi Rotavapor R-3) under reduced pressure (Vacuum Pump, Büchi V-850 Vacuum Controller) at 40 °C. Then, the extracted ZEN was dissolved in 500 μL methanol-distilled water mixture (50 *v*/*v%*) and the ZEN content of the produced solution was analyzed by HPLC-FLD (see details in Section 4.5). Extraction of ZEN from beer was tested at higher and lower mycotoxin concentrations (i.e., 100–600 nM) as well. These experiments indicated that the recovery of ZEN was constant regardless of its concentration.

### 4.5. HPLC Analyses

Quantitation of ZEN and ZELs was performed by the method of Visconti and Pascale [31] with minor modifications. The analyses applied an HPLC pump (model 510, Waters, Milford, MA, USA) to drive eluent through an injector (Rheodyne 7125) supplied with a 20-μL sample loop onto a SecurityGuard Catridge (C18 4.0 × 3.0 mm, Phenomenex, Torrance, CA, USA) coupled to a Kinetex XB-C18 analytical column (250 × 4.6 mm, 5 μm particle size, Phenomenex, Torrance, CA, USA). The mobile phase contained acetonitrile (VWR), distilled water, and methanol (VWR): 46:46:8 *v*/*v%*. After injecting 20 μL samples, the isocratic elution was performed at room temperature with a 1 mL/min flow rate. Since the examined mycotoxins are fluorescent molecules [27,32], ZEN and ZELs were quantified by a fluorescent detector (Jasco FP-920; λ_ex_ = 274 nm, λ_em_ = 440 nm). Data were recorded and evaluated using the Millennium Chromatography Manager (Waters, Milford, MA, USA), which also controlled the HPLC pump.

### 4.6. Testing the Influence of BBP on the Color and Polyphenol Content of Corn Beer

Since BBP is not a selective binder of ZEN and ZELs, it is reasonable to hypothesize that not only mycotoxins, but also other compounds, may be extracted from beer by BBP. To test this effect of BBP, the color and the polyphenol content of corn beer samples were examined applying the previously described methods [33]. UV-Vis spectroscopic analyses were performed applying the HALO DB-20 (Dynamica) spectrophotometer. Color of the degassed beer was evaluated based on its absorbance at 430 nm, using the following equation [33]:

(3) $$C=A_{430}\times f\times 25$$

where *C* denotes the color, *A*_430_ is the absorbance of samples at 430 nm, and *f* is the dilution factor.

During the investigation of the total polyphenol content of beer, a 1 mL fraction of degassed beer sample was added to 0.8 mL of CMC/EDTA reagent (2% of carboxymethylcellulose sodium salt and 0.4% of ethylenediaminetetraacetic acid disodium salt in water) and vortexed. Then, 50 μL of ferric reagent (3.5% ammonium ferric citrate in water) and 50 μL of ammonia reagent (pure concentrated ammonia diluted with two volumes of water) were added to the mixture. After making it up to a 2.5 mL final volume, the solution was mixed again. Samples were incubated for 10 min at room temperature, the optical density was measured at 600 nm, and polyphenol content was determined applying the following equation [33]:

(4) $$P=A_{600}\times 820$$

where *P* denotes the total polyphenol content (mg/L), while *A*_600_ is the absorbance of samples at 600 nm.

### 4.7. Statistics

Mean ± SEM values were derived from at least three independent experiments. During the statistical analyses of data, a One-Way ANOVA test was applied (using IBM SPSS Statistics, Version 21). The level of significance was set as *p* < 0.01.

## Figures and Tables

**Table 1:** Isotherm parameters (±SEM) obtained by the graphical application of Langmuir and Freundlich models (Equations (1) and (2)) for the extraction of ZEN and ZELs by BBP in water.

Model		ZEN	α-ZEL	β-ZEL
Langmuir	*Q*_0_ (mg/g)	3.11 (±0.86)	5.28 (±1.41)	3.31 (±0.75)
	*K_L_* (L/mg)	0.60 (±0.25)	0.75 (±0.25)	0.47 (±0.15)
	*R* ^2^	0.982	0.997	0.993
Freundlich	*K_F_* (mg/g)(L/mg)^1/n^	1.16 (±0.07)	2.72 (±0.16)	1.04 (±0.01)
	1/*n*	0.79 (±0.10)	0.87 (±0.05)	0.76 (±0.02)
	*R* ^2^	0.967	0.995	0.999

**Table 2:** Testing the reactivation and the reusability of BBP as a mycotoxin binder: Extraction of ZEN by BBP and elution of the mycotoxin from BBP by ethanol-water mixture.

Number of Applications	Procedure Performed	ZEN (%) in the Buffer (A) or the Eluent (B, C)	∑ (%)
1st application of the polymer	A: After extraction with BBP	8.0 ± 0.3	99.8
	B: After the 1st elution	85.8 ± 1.6	
	C: After the 2nd elution	6.0 ± 0.7	
2nd application of the polymer	A: After extraction with BBP	7.1 ± 0.2	98.3
	B: After the 1st elution	84.8 ± 1.5	
	C: After the 2nd elution	6.4 ± 0.8	
3rd application of the polymer	A: After extraction with BBP	7.4 ± 0.4	-

A: Percent of ZEN (10 μM) remaining in 1.5 mL sodium acetate buffer (pH 5.0), after its incubation with 10 mg BBP for 40 min at 25 °C. B: Percent of ZEN recovered in the ethanol-water mixture, after the first elution from the polymer with 1.5 mL 50 *v*/*v%* ethanol for 10 min at 25 °C. C: Percent of ZEN recovered in the ethanol-water mixture, after the second elution from the polymer with 1.5 mL 50 *v*/*v%* ethanol for 10 min at 25 °C.
